# Review of "Electrical Impedance Tomography" Edited by David S. Holder

**DOI:** 10.1186/1475-925X-4-27

**Published:** 2005-04-15

**Authors:** Ge Wang

**Affiliations:** 1 Department of Radiology University of Iowa 200 Hawkins Drive Iowa City, IA 52242, USA

As implied by its name, electrical impedance tomography (EIT) is to reconstruct an impedance distribution in an object of interest from electrical measurement on the boundary of the object. Because of its non-invasiveness, portability and practical utilities, EIT has been actively studied since 1970s and led to many useful and inspiring results. There are already two books on this subject. One is a general textbook published in 1990 [1]. The other is a monograph from a conference on biomedical applications in 1992 [2]. Given the steady development in EIT theory, technology and applications over the past decade, the book under review is indeed overdue to provide an overview of such a fascinating field.

This book consists of four parts on algorithms, hardware, applications, and directions, along with two introductory appendices on bioimpedance and biomedical EIT, respectively. The first and only chapter in the first part of the book formulates the problem and describes associated reconstruction methods. The second chapter addresses instrumentation issues, which is the only chapter in the second part. The third through seventh chapters form the third part to cover various biomedical applications, including imaging of the thorax, brain, breast, gastrointestinal tract, hyperthermia, intra-pelvic venous congestion, and so on. Finally, as the fourth part the subsequent six chapters are respectively dedicated to three unique modes of EIT (magnetic induction tomography, magnetic resonance EIT, industrial process tomography) and professional perspectives from three leading groups (Sheffield and Oxford Brookes Universities in UK as well as Rensellaer Polytechnic Institute in USA). The two appendixes are very educational for non-experts to appreciate the ideas and features of EIT. There are 141 formulas and 183 figures in the book. While generally speaking it is in high quality, the formatting and compiling work could have been done better. For example, some formulas carry no indexes; a number of figures are not given captions; full bibliographic information is not given to few cited references; and there are some minor typos.

All the chapters are well written by established authorities, whose opinions on the future directions are also included. There is no doubt that the book serves its purpose well, which I read with pleasure and satisfaction. Clearly, the book provides a solid foundation to understand the big picture, technical contents and open problems of EIT and to prepare a mathematician, an engineer, or a technologist for research and development in various aspects of EIT, and a biomedical researcher or a clinician for applications of EIT techniques. Nevertheless, in my opinion, the theoretical and algorithmic aspects could have been covered more systematically and more thoroughly. For example, the book would be even more valuable if a more comprehensive review has been presented on Maxwell's equations, more rigorous discussions been given on existence, uniqueness and stability of the EIT solution, and representative reconstruction algorithms described in more details.

An insightful comment on the current status of this field was made by Dr. Holder, the editor of this book, that "*it doesn't clearly work, so we can reap the fruits of its images, or not work, so we can change direction; it usually almost works, which is an incitement to redouble our efforts*." I believe that many active researchers who handle highly ill-posed problems may more or less share his feeling, and that is exactly why these kinds of problems are attractive to motivated investigators. The advice and lessons given in the book are numerous, covering improvement of data acquisition, regularization of algorithms, incorporation of *a priori *knowledge, avoidance of tweaking algorithmic parameters, use of different meshes, simulation of realistic noise and errors, fusion of complementary modalities, *etc*. Interestingly, I notice a substantial similarity between EIT and some molecular imaging modalities, such as fluorescence/bioluminescence tomography [3,4]. The interaction between these two research areas should be mutually beneficial. From that perspective, this book is helpful for further research not only on EIT but also on other highly ill-posed inversion problems; for example, fluorescence/bioluminescence tomography.

With the momentum of the EIT research, it seems very likely that this technology will gain much wider acceptance in clinical scenarios, along with other emerging biomedical imaging methods. Hence, I highly recommend this masterpiece for imaging scientists and engineers who are interested in EIT, and sincerely suggest that all those who are involved with tomographic imaging and noninvasive testing would be benefited by reading such an excellent text, even just some chapters.
